# Brain Iron Homeostasis: A Focus on Microglial Iron

**DOI:** 10.3390/ph11040129

**Published:** 2018-11-23

**Authors:** Israel C. Nnah, Marianne Wessling-Resnick

**Affiliations:** Department of Genetics and Complex Diseases, Harvard TH Chan School of Public Health, Boston, MA 02115, USA; innah@hsph.harvard.edu

**Keywords:** Alzheimer’s disease, neuroinflammation, neurodegeneration, cytokines, neuroimmune responses

## Abstract

Iron is an essential trace element required for important brain functions including oxidative metabolism, synaptic plasticity, myelination, and the synthesis of neurotransmitters. Disruptions in brain iron homeostasis underlie many neurodegenerative diseases. Increasing evidence suggests that accumulation of brain iron and chronic neuroinflammation, characterized by microglia activation and secretion of proinflammatory cytokines, are hallmarks of neurodegenerative disorders including Alzheimer’ s disease. While substantial efforts have led to an increased understanding of iron metabolism and the role of microglial cells in neuroinflammation, important questions still remain unanswered. Whether or not increased brain iron augments the inflammatory responses of microglial cells, including the molecular cues that guide such responses, is still unclear. How these brain macrophages accumulate, store, and utilize intracellular iron to carry out their various functions under normal and disease conditions is incompletely understood. Here, we describe the known and emerging mechanisms involved in microglial cell iron transport and metabolism as well as inflammatory responses in the brain, with a focus on AD.

## 1. Introduction

The brain is among the most metabolically active organs in the body and accounts for at least 20% of the body’s energy consumption. Accordingly, an adequate supply of iron is necessary to sustain its high-energy needs [1,2,3,4]. Our understanding of the role of iron in normal brain function has improved tremendously over the last decade, with much attention directed towards deciphering the cellular and molecular cues that guide brain iron transport and metabolism. These efforts have described the essential roles of iron as a co-factor for several physiological processes including oxidative metabolism, myelination, and the biosynthesis of neurotransmitters [5,6,7]. However, excess iron is known to contribute to homeostatic dysregulation due to oxidative stress and has been linked to a number of neurological disorders. Being redox active, iron exists in both ferrous (Fe^2+^) and ferric (Fe^3+^) forms and constantly cycles between the two states. Under aerobic conditions, this redox cycling has the potential to generate highly reactive free radicals through Fenton chemistry, resulting in oxidative stress and damage to macromolecules. Thus, the metal is directly implicated in the disease known as neurodegeneration with brain iron accumulation (NBIA), and, in addition to other trace elements implicated in neurodegeneration, including copper [8], manganese [9], and zinc [10], increasing evidence support iron’s role in several other sporadic or genetic neurodegenerative disorders such as Alzheimer’s disease (AD), Parkinson’s disease (PD), Huntington’s disease (HD), amyotrophic lateral sclerosis (ALS), and multiple sclerosis (MS) [11,12,13,14].

Microglia make up 5 to 12% of the population of cells found in mouse brain and about 0.5 to 16% of those in the human brain [15,16]. These resident macrophages are largely involved in immune responses and, depending on the stimuli, they can adopt a range of pro- or anti-inflammatory states to help maintain the integrity of the neural environment [17,18,19]. In addition to their roles in the neuroinflammatory response, microglia participate in neurogenesis [19,20], shaping and maintaining synaptic density and connectivity in the adult and developing central nervous system (CNS) [16,21,22,23,24], oligodendrocyte differentiation [25], synaptic pruning [26], and myelin repair [16]. Microglia require iron as a co-factor to carry out all of these varied functions [27]. Over the years, multiple studies have reported the roles these immune cells play in brain iron homeostasis [1,27,28]. This review will examine the influence of brain iron on microglial metabolism and corresponding inflammatory responses under normal and neurodegenerative conditions, with a particular focus on AD.

## 2. Brain Iron

Brain iron levels are tightly regulated to ensure the normal function of the CNS [29,30]. The major route of iron acquisition begins with intestinal absorption, as dietary Fe^3+^ is reduced to Fe^2+^ by duodenal cytochrome B (DcytB) at the apical surface of enterocytes [31]. Divalent metal transporter-1 (DMT1) imports Fe^2+^ into the intestinal cells, while the iron exporter ferroportin (Fpn) mediates its exit across this epithelial barrier. On the serosal side, the multicopper ferroxidases ceruloplasmin and/or hephaestin oxidize Fe^2+^ to Fe^3+^, thereby promoting its binding to the iron carrier protein transferrin (Tf) [32]. Dietary absorption of iron is tightly regulated to respond to the body’s iron needs, such that uptake is enhanced by iron deficiency but reduced under iron-loading conditions [29]. Thus, iron supplied to the brain from the diet reflects nutrient demands, while limiting the potential for excessive accumulation.

Once in the circulation, the entry of iron into the brain from the blood is controlled by the blood–brain barrier (BBB) [33]. The BBB is formed by brain microvascular endothelial cells (BMVECs), pericytes, and astrocytes [33,34,35]. Tf-bound iron circulating in the blood outside the CNS cannot cross the BBB directly, and, therefore, iron must enter the brain through BMVECs in a multi-step transcellular pathway. Binding of Tf to Tf receptors (TfR) at the lumen of the brain microvasculature facilitates iron uptake via receptor-mediated endocytosis [30,34,36]. The subsequent fate of the Tf–TfR complex within brain endothelial cells is not entirely clear, and exactly how iron is released to the brain remains controversial. The transcytosis model suggests that the ligand–receptor complex traverses the cell, such that Tf is released to the interstitium. However, how Tf might dissociate from its receptor at the abluminal membrane remains unexplained. An alternative model is that iron is released to the cytoplasm of BMVECs after receptor-mediated endocytosis of Tf. The endocytic uptake pathway for iron is much better understood and involves the release of Fe^3+^ from Tf in the acidic endosomal environment, its reduction to Fe^2+^, and DMT1-mediated export from the endosome [29]. However, whether BMVECs express DMT1 or if its function is required for entry of iron into the brain is unclear, since different groups have reported conflicting data [37,38,39,40,41,42]. An alternative membrane transport mechanism could involve transient receptor potential mucolipin-1 (TRPML1) channels which function in the release of iron from endolysosomal compartments [43]. A recent study has shown that loss of TRPML1 in mice promotes dysregulation of brain homeostasis and decreased myelination, suggesting a potential role in brain iron uptake [44]. Regardless of which transporter is responsible for iron’s exit from endocytic compartments, the metal would then be utilized for metabolic purposes by the endothelial cells, stored in endothelial cell ferritin (Ftn), or released to the brain via Fpn [45]. Re-oxidation of Fe^2+^ to Fe^3+^ and subsequent incorporation into apo-Tf would provide for its circulation in the brain [46,47,48]. It is possible that hepcidin, which is produced by the brain endothelium, plays a role in regulating this process. An in-depth review of iron uptake into BMVECs and its release has been published elsewhere [33].

It is important to note that the amount of Tf in the brain interstitial fluid is thought to be much lower than the levels in the systemic circulation, while non-Tf-bound iron (NTBI) levels may be quite high [49]. Thus, although Tf is apparently involved in moving iron across the BBB, there is some evidence to suggest that Tf-iron-binding sites may become saturated in the brain, such that NTBI is a major source of iron delivery to neurons and other cells in the brain. Another alternative source of iron is ferritin which plays an important role in brain iron homeostasis. In fact, genetic loss of ferritin function leads to brain iron dyshomeostasis [11,50,51,52]. The brain may acquire ferritin exogenously by transcellular transport across the BBB, or it may be produced by endothelial cells and released upon demand [53]. Other endogenous sources of brain ferritin are possible, including its synthesis by microglia [28]. The ferritin pathway of iron delivery is particularly important for mouse oligodendrocytes and their function in myelination and neuronal repair. These express the ferritin receptor Tim-2, a member of the T cell immunoglobulin and mucin domain family, and specifically take up ferritin [6,54]. In humans, the transferrin receptor may bind to and mediate the internalization of ferritin [55,56].

## 3. Functions of Iron in the Brain

**Figure 1:**
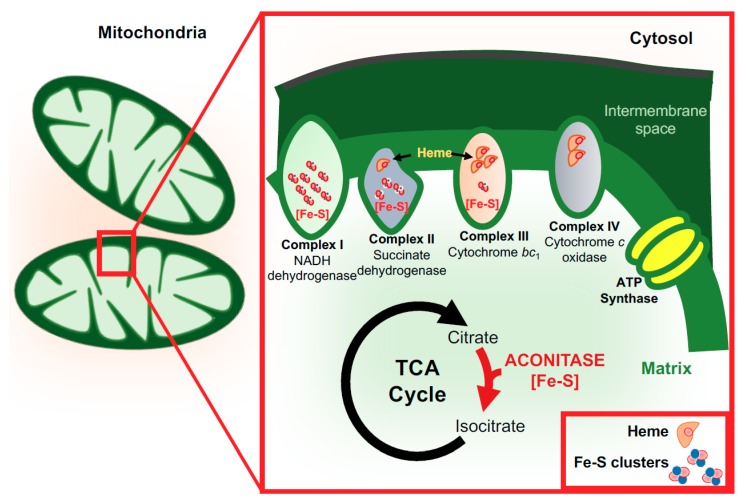
Iron and mitochondrial function. The mitochondrial electron transport chain contains multiple iron–sulfur clusters and heme-containing proteins necessary for ATP synthesis. NADH dehydrogenase (complex I) contains eight Fe–S clusters, succinate dehydrogenase (complex II) contains three Fe–S clusters and one heme moiety, while complex III (cytochrome bc1) contains one Fe–S cluster and several heme groups vital for its functions. Complex IV (cytochrome c oxidase) also contains two heme moieties. Aconitase, a key enzyme that catalyzes the stereo-specific isomerization of citrate to isocitrate through cis-aconitate in the tricarboxylic acid (TCA) cycle contain Fe–S clusters.

Iron plays an indispensable role during ATP production by serving as a cofactor for cytochromes and iron–sulfur complexes of the oxidative chain [57]. The major substrate for brain energy production is glucose which becomes fully oxidized; ketone bodies can fulfill energy needs under some conditions. The brain consumes nearly 20% of the body’s energy, although representing only about 2% of its weight. About 75–80% of the energy supports neuronal activity, with the remainder utilized to maintain the “housekeeping” functions of astrocytes, oligodendrocytes, and microglia [4]. Neuronal energy needs represent both axonal and synaptic signaling, but the majority is utilized post-synaptically [58]. The mitochondrial function must provide this supply of ATP with the iron requirements to support oxidative phosphorylation, as shown in Figure 1.

Oligodendrocytes, which are responsible for producing myelin, also require high amounts of ATP [59]. Not only do many of the enzymes involved in ATP production require a supply of iron, but also pathways for cholesterol and fatty acid synthesis necessary for myelination are iron-dependent. Some of the enzymes involved in this pathway include NADH dehydrogenase, HMG-CoA reductase, succinate dehydrogenase, dioxygenase, and glucose-6-phosphate dehydrogenase, all of which are abundant in oligodendrocytes compared to other cell types of the CNS [59]. The need for an adequate supply of iron during myelination is reflected in the results of animal studies demonstrating that dietary iron restriction reduces the amount and composition of myelin during gestation and early post-natal periods [60,61].

Neurotransmitters serve as means of communication between neurons. The process of this communication includes biosynthesis and transport of neurotransmitters, packaging of neurotransmitters into vesicles for storage and controlled release, and binding of neurotransmitters to receptors on post-synaptic neurons with induction of cellular responses. The role of iron in each of these processes has been reviewed extensively, particularly in the case of monoamine neurotransmitters such as dopamine and serotonin that are involved in the regulation of cognitive processes including emotion and arousal behaviors [1,62,63,64,65]. For example, the synthesis of monoamine neurotransmitters depends on tyrosine hydroxylase which is an iron-requiring enzyme [66,67]. The activity of this enzyme is significantly reduced in patients suffering from PD with compromised brain iron homeostasis [67]. Iron deficiency further alters the functioning of the dopaminergic systems, with specific effects on dopamine D_1_ and D_2_ receptors [1,68]. Studies carried out by Youdim and colleagues demonstrated that the densities of dopamine D_2_ receptors are significantly lower in the striatum of rats deficient in iron [69,70,71]. Also, microdialysis studies demonstrated an elevation of extracellular dopamine in the striatum of iron-deficient rats and the return to basal levels when brain iron content and iron status returned to normal [72]. In the case of serotonin, tryptophan hydroxylase carries out the rate-determining step in the synthesis of this neurotransmitter and can be inhibited by iron chelators [66,73]. Another neurotransmitter whose biosynthesis is compromised under iron-deficient conditions is gamma-aminobutyric acid (GABA), the main inhibitory neurotransmitter in the CNS. Iron deficiency is associated with significant reduction in the activity of glutamate dehydrogenase and GABA transaminase, key enzymes responsible for the synthesis and degradation of GABA [74,75].

## 4. Microglia and Iron

Microglial activation in response to pro- and anti-inflammatory stimuli is often characterized either as classical M1 or as alternative M2, similar to the nomenclature used for systemic macrophages [76,77]. M1 activation is pro-inflammatory and neurotoxic and primarily induced through the activation of toll-like receptor (TLR) and interferon gamma (IFN-γ) signaling pathway [19]. M1 microglia synthesize and secrete pro-inflammatory cytokines and chemokines such as tumor necrosis factor-alpha (TNF-α), some members of the interleukin family of cytokines interleukin-6 (IL-6), interleukin 1-beta (IL-1β), interleukin-12 (IL-12), and C-C Motif Chemokine Ligand 2 (CCL2). In this reactive state, microglia also express inducible nitric oxide synthase (iNOS), which converts arginase into nitric oxide [19]. Accumulation of nitric oxide increases the toxic effects of glutamate and consequently potentiates N-methyl-D-aspartate (NMDA) receptor-mediated neurotoxicity [19,78,79].

In the M2 state, microglia release anti-inflammatory cytokines such as interleukin-10 (IL-10) and transforming growth factor-beta (TGF-β). M2 microglia also induce arginase 1 to promote the conversion of arginine into polyamines [80]. These cells can secrete insulin-like growth factor I (IGF-I), fibroblast growth factor (FGF), and neurotrophic factors including nerve growth factor (NGF) and brain-derived neurotrophic factor (BDNF), in the effort to resolve inflammation and promote synaptic plasticity [19].

The use of the terms “M1 versus M2” oversimplifies a complex process for microglial activity, since transcriptome studies have revealed that activation is quite variable and context-dependent [18,81]. Indeed, microglia adopt a homeostatic (M0) state under normal conditions in the CNS, and their transcriptome profile reflects their immunosurveillance activities in this state [18,81,82,83]. Conversely, microglia can express both neurotoxic and neuroprotective factors under disease conditions [19,81,84].

One prominent hallmark of neuroinflammation is the activation and increased acquisition of extracellular iron and subsequent downregulation of iron-interacting proteins, causing the intracellular sequestration of iron [13]. Systemically, such innate immune responses are orchestrated to deprive invading pathogens of iron, necessary for their survival [85]. This “iron withdrawal” phenomenon could play a similar role in the brain to reduce the metal’s availability. However, accumulation of intracellular iron is associated with neuronal degeneration that underlies most neurological disorders [86], and microglial secretion of the inflammatory cytokines TNF-α and IL-1β enhances neuronal iron uptake [87]. In turn, these pro-inflammatory mediators have been shown to strongly influence microglia iron transport and metabolism [13,88,89,90].

Microglial cells interact with both Tf bound-iron (TBI) and NTBI [91], and pathways for each transport substrate have been characterized [28]. For NTBI uptake, an endogenous cell surface ferrireductase reduces Fe^3+^ to Fe^2+^ for uptake by DMT1 in a pH-dependent manner at the cell surface. TBI is taken up by endocytosis of the Tf–TfR complex; after the release of iron in the acidic milieu of the endosome, it is translocated into the cytosol by DMT1 or other transporters, as described above [92].

**Figure 2:**
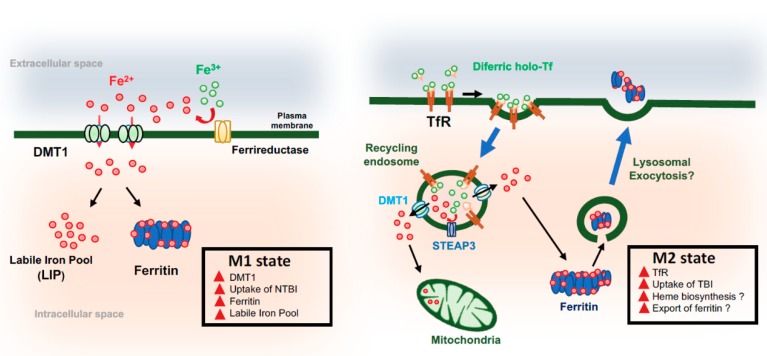
Iron trafficking and microglial cell polarization. (**Left**) Pro-inflammatory stimuli upregulate the expression of divalent metal transporter-1 (DMT-1) and the uptake of non-Tf-bound iron (NTBI). These effects are associated with increased labile iron and an expanded pool of ferritin. These changes reflect M1 polarization. (**Right**) Anti-inflammatory stimuli increase transferrin receptor (TfR) levels to upregulate Tf-bound iron (TBI) uptake by receptor-mediated endocytosis. In recycling endosomes, the low pH promotes the release of Fe^3+^ for ferrireduction, most likely by STEAP3. Fe^2+^ may be released into the cytosol through DMTI or another channel for use in mitochondria to promote heme production. We speculate that, under anti-inflammatory conditions, microglia may release ferritin (Ftn)-bound iron through lysosomal exocytosis to help oligodendrocyte function and neuronal repair.

Early studies of rat microglia raised the idea that microglial polarization and iron uptake are coordinated [89]. More recently, our group has shown that microglial iron transport pathways are differentially active in response to pro- and anti-inflammatory stimuli at both the transcript and the protein levels. Pro-inflammatory mediators increase the uptake of NTBI and expand the ferritin storage pool. These changes reflect the upregulation of both DMT1 and ferritin [28]. The uptake of NTBI by microglia would limit free extracellular iron and reduce potentially damaging reactive oxygen species (ROS) in the neural environment. In this M1 pro-inflammatory state, microglial cells also have increased glycolysis, with extracellular acidification supporting changes in the microenvironment favoring NTBI uptake by the pH-dependent transporter DMT1. Inflammatory mediators also reduce oxidative respiration, induce heme oxygenase-1, and diminish the levels of intracellular heme. These changes are associated with increased intracellular “labile iron”, suggesting that microglia can sequester both intracellular iron released by heme catabolism and extracellular iron taken up by DMT1. In contrast, anti-inflammatory IL-4 increases the expression of TfR to promote the uptake of TBI [28]. It is possible that this shift in iron transport is associated with the release of ferritin stores by M2 microglia to support the regeneration of neurons and the activity of oligodendrocytes. On the basis of these data, we propose a model by which microglia actively modify transport pathways and metabolism in response to the iron status of their environment (Figure 2).

## 5. Microglia Activation and Alzheimer’s Disease

Alzheimer’s Disease (AD) is a neurodegenerative disorder and the most common form of dementia involving the progressive loss of substantive cortical and hippocampal neurons over time [93,94]. This disorder is characterized by extracellular deposition of amyloid beta (Aβ) in senile plaques and intraneuronal accumulation of hyperphosphorylated tau proteins. These events lead to neuronal and synaptic loss, chronic inflammation, and oxidative stress [19,89,95,96].

Genetic studies of familial Alzheimer’s disease (FAD) have demonstrated that mutations in the amyloid precursor protein and in components of the gamma-secretase complex generate Aβ1-42 which can misfold and aggregate [19,93,97]. The more common sporadic form (SAD) of the disease is largely associated with aging. Although the pathophysiological mechanisms that underlie the role of aging in the onset of AD is poorly understood, accumulating evidence indicates that the onset of SAD is closely associated with brain iron and oxidative stress, both of which increase with age [95,98,99,100,101,102,103]. In AD, the observation that iron is present in local areas of neuronal cell death further supports the metal theory of dementia which proposes that iron promotes neurodegeneration [104]. Furthermore, as brain microglia are implicated in iron handling, it has been shown that iron accumulates in microglial cells that cluster around amyloid plaques in AD mouse models and post-mortem brain tissues of AD patients [105].

Iron is a key player during the induction of oxidative stress because of its function as a redox-active transition metal [13,96,106]. Indeed, the levels of damaging ROS are significantly higher in the AD brain compared to healthy control brains [96,107]. Importantly, several studies have reported that, by promoting neurotoxic oligomerization of Aβ peptides and tau tangles [94,108], oxidative stress potentiates the activation of microglia [96,109,110]. Whether these events advance or hinder the disease is subject to active debate. For example, while anti-inflammatory activities of microglia would appear to be beneficial, some studies have reported that prolonged stimulation of microglia with Aβ peptides provokes chronic inflammation [111].

Microglial cells express multiple receptors including CD36, TLR2, TLR4, and TLR6 [111,112,113], all of which can bind Aβ and induce pro-inflammatory effects. The sporadic form of AD is associated with genetic variants of triggering receptor expressed on myeloid cells 2 (TREM2) [114]. TREM2 is an immune transmembrane glycoprotein receptor expressed in microglia that interacts with phospholipids, apoptotic cells, and lipoproteins [115,116,117,118]. These variants, as well as the loss-of-function mouse models of AD, appear to limit microglial proliferation around Aβ plaques, causing increased plaque buildup and disease progression [119,120,121,122]. Why defective TREM2 function or expression impacts microglia responses to AD lesion is still incompletely understood. The role of TREM2 and other immune receptors identified as risk factors, including CD33, have been reviewed elsewhere [19].

SAD is also associated with the polymorphism of apolipoprotein E (apoE), a lipid-binding protein involved in lipid metabolism [123,124,125]. The apoE4 allele is strongly associated with an increased risk of AD, while apoE2 serves a protective role [123]. Microglia produce apoE, which has been shown to moderate the inflammatory response while enhancing the phagocytosis of Aβ aggregates by microglia [124,125,126,127,128]. However, in carriers of apoE4, increased levels of ferritin have been reported in the cerebral spinal fluid, suggesting that iron metabolism is altered in these individuals to promote increased iron retention [129]. This evidence reinforces the concept that increased brain iron adversely affects patients with AD. In fact, patients with HFE-associated hemochromatosis are subject to earlier onset of AD [130]. Since there is clinical evidence that iron chelation is beneficial to AD patients [131,132], the relationship between microglia, iron, and neurodegeneration appears to be well worth exploring.

## 6. Concluding Remarks

Understanding the role of iron in chronic inflammatory responses elicited by microglia is essential for finding new therapeutic strategies to treat neurodegenerative diseases. Although a substantial amount of effort has been put into deciphering the molecular network directly involved in brain iron metabolism, we still must pursue an in-depth understanding of how specific brain cells accumulate and use iron to carry out their various functions, both in normal and in disease conditions.

## Abbreviations

Alzheimer’s disease	AD
familial Alzheimer’s disease	FAD
sporadic Alzheimer’s disease	SAD
neurodegeneration with brain iron accumulation	NBIA
Parkinson’s disease	PD
Huntington’s disease	HD
amyotrophic lateral sclerosis	ALS
multiple sclerosis	MS
amyloid beta	Aβ
transferrin	Tf
Tf-bound iron	TBI
transferrin receptor	TfR
non-transferrin-bound iron	NTBI
divalent metal transporter-1	DMT1
labile iron pool	LIP
central nervous system	CNS
Blood–brain barrier	BBB
brain microvascular endothelial cell	BMVEC
transient receptor potential mucolipin-1	TRPML1
ferritin	Ftn
reactive oxygen species	ROS
gamma-aminobutyric acid	GABA
inducible nitric oxidase	iNOS
N-methyl-D-aspartate	NMDA
adenosine triphosphate	ATP
3-hydroxy-3-methyl-glutaryl-coenzyme A	HMG-CoA
tricarboxylic acid	TCA
interferon gamma	IFN-γ
toll-like receptor	TLR
tumor necrosis factor-alpha	TNF-α
interleukin-6	IL-6
interleukin 1-beta	IL-1β
interleukin-12	IL-12
interleukin-10	IL-10
C-C motif chemokine ligand-2	CCL2
transforming growth factor-beta	TGF-β
insulin-like growth factor-1	IGF-1
fibroblast growth factor	FGF
nerve growth factor	NGF
brain-derived growth factor	BDNF
toll-like receptor 2	TLR2
toll-like receptor 4	TLR4
toll-like receptor 6	TLR6
cluster of differentiation 33	CD33
cluster of differentiation 36	CD36
triggering receptor expressed on myeloid cells 2	TREM2
apolipoprotein E	apoE
apolipoprotein E4	apoE4
apolipoprotein E2	apoE2
